# A general method for analyzing arbitrary planar negative-refractive-index multilayer slab optical waveguide structures

**DOI:** 10.1038/s41598-020-72017-3

**Published:** 2020-09-11

**Authors:** Yaw-Dong Wu

**Affiliations:** Electronic Engineering Department, National Kaohsiung University of Science and Technology, Kaohsiung, Taiwan

**Keywords:** Optics and photonics, Metamaterials

## Abstract

In this paper, a general method for analyzing arbitrary planar negative-refractive-index (NRI) multilayer slab optical waveguide structures was proposed. Some degenerated examples were introduced to prove the accuracy of the proposed method. The analytical and numerical results show excellent agreement. The method can also be degenerated to analyze arbitrary planar conventional optical waveguide structures. Based on this general method, the analysis and calculation of any kinds of planar NRI slab optical waveguide structures and planar conventional optical waveguide structures can be achieved easily.

## Introduction

The left-handed material with double-negative media ($\varepsilon$ < 0, $\mu$ < 0) is different from the right-handed material with double-positive media ($$\varepsilon > 0,\begin{array}{*{20}c} {} \\ \end{array} \mu > 0$$). It has been proposed theoretically and experimentally by Veselago and Shelby et al., respectively^1,2^. The NRI media possess simultaneously the negative dielectric permittivity and permeability^3,4^. Therefore, the NRI media is different from right-handed material with double-negative media. Recently, metamaterial has attracted a lot of attention^5–14^. Many applications of metamaterial have been proposed in various fields, such as antennas, perfect absorber, super lens, invisibility cloaks, optical sensors, phase modulators, and phase holography^5,15–33^. Some numerical and experimental results of metamaterial have also been proposed^34–40^. In the past, all–optical devices based on the conventional nonlinear optical waveguide structures have been proposed, since the spatial solitons can propagate a long distance without changing their spatial shapes^41–46^. In the past, most papers just focused on the study of the properties at the interface between the right-handed material and the metamaterial^47–53^. The TE/TM surface polarizations propagating along the interface between a linear metamaterial and different types of conventional right-handed material were studied. The three-layer metamaterail waveguide with linear cladding and substrate had been discussed^54,55^. In our previous work^56^, we proposed a special case of the NRI multilayer slab optical waveguide structures with only the Kerr-type nonlinear cladding. The analyzed processes of the proposed structure are relatively simple compared to that of this manuscript. When all layers of the proposed NRI slab optical waveguide structure are the Kerr-type nonlinear media, the analyses processes will become very complicated and difficult. The difficulty lies in the derivation of the mathematical model and the verification of numerical analyses and simulations. To the best of my knowledge, a general method for analyzing arbitrary NRI slab optical waveguide structures has not been proposed before. This paper gives detailed modal analyses of TE-polarized waves in the NRI multilayer slab waveguide structure with all Kerr-type nonlinear layers. The theoretical results and the numerical results show excellent agreement. The method can also be used to investigate and to analyze the distribution of TE electrical field in the Kerr-type nonlinear NRI multilayer slab optical waveguide structures. To prove the accuracy of the proposed general method for analyzing arbitrary NRI multilayer slab optical waveguide structures, a theoretically degenerated example was introduced. Therefore, the method can provide simultaneously to analyze two different kinds of waveguide structures. One is the nonlinear NRI multilayer slab optical waveguide structure and the other is the linear NRI multilayer slab optical waveguide structure. Based on this general method, the analysis and calculation of any kinds of NRI multilayer slab optical waveguide structures can be achieved easily.

## Analysis

**Figure 1:**
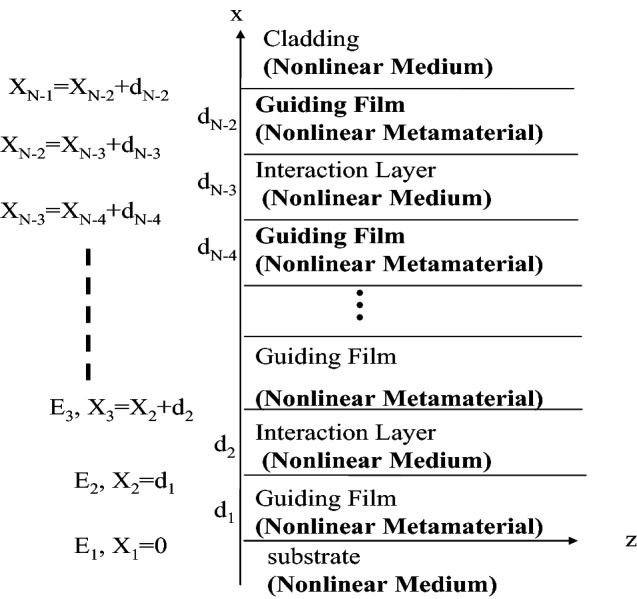
The proposed NRI multilayer slab optical waveguide structure with all Kerr-type nonlinear layers.

In general, a transfer matrix approach^57^ can be used to analyze the conventional multilayer slab optical waveguide structures. However, it cannot be used to analyze the case of the NRI multilayer slab optical waveguide structure with all Kerr-type nonlinear layers, proposed in this manuscript. When all layers of the proposed NRI slab optical waveguide structure are the Kerr-type nonlinear media, the analytical solutions are very complicated because they contain the Jacobian elliptic functions. It is very difficult to obtain the exact solutions. In this paper, the modal theory^58^ was used to derive the formulae of the electric field distributions of the proposed NRI multilayer slab optical waveguide structure with all Kerr-type nonlinear layers, as shown in Fig. 1. The multilayer optical waveguide structure is composed of the guiding films ((N-1)/layers), the interaction layers ((N − 3)/2layers), the cladding layer, and the substrate layer. The total number of layers is N (N = 3, 5, 7,…). The *d*_*j*_ and *n*_*j*_ are used to denote the width and the refractive index of the *j*th layer, respectively. The nonlinear cladding and substrate layer are assumed to extend to infinity in the + x and − x directions, respectively. The major significance of this assumption is that there are no reflections in the x direction to be concerned with, expect for those occurring at interfaces. For the simplicity, the TE waves are choosing to propagate along the z direction. The wave equation in the *j*-th layer can be written as:

1 $$ \nabla^{2} E_{yj} = \frac{{n^{2}_{j} }}{{c^{2} }}\frac{{\partial^{2} E_{{{\text{yj}}}} }}{{\partial t^{2} }}\begin{array}{*{20}c} , & {j = 0,1,2, \ldots ,N - 1} \\ \end{array} $$

with the solutions of the form:

2 $$ E_{{{\text{yj}}}} \left( {x,z,t} \right) = E_{j} \left( x \right)\exp [i\left( {\omega t - \beta k_{o} z} \right)] $$

where β is the effective refractive index, ω is the angular frequency, and $k_{0}$ is the wave number in the free space. For the Kerr-type nonlinear medium, the square of the refractive index $n_{j}^{2}$ in the nonlinear NRI slab guiding films, nonlinear interaction layer, nonlinear cladding, and nonlinear substrate can be expressed as^48^:

3 $$ n_{j}^{2} = \varepsilon_{j} \mu_{j} + \alpha_{j} \left| {E_{j} \left( x \right)} \right|^{2} \begin{array}{*{20}c} , & {j = 0,1,2, \ldots ,N} \\ \end{array} - 1 $$

where $\varepsilon_{j}$ and $\mu_{j}$ (j = 1, 3,…, N − 2) are the negative dielectric permittivity and permeability in the nonlinear NRI slab guiding layer, respectively, and The rest layers are the positive dielectric permittivity and permeability. The α_j_ is the nonlinear coefficient of the j-th layer Kerr-type nonlinear medium. By substituting Eqs. (2) and (3) into the wave equation Eq. (1), the transverse electric fields can be solved in each layer. The transverse electric fields in each layer can be expressed as:

4 $$ \left[ {\frac{{d^{2} }}{{dx^{2} }} + q_{j}^{2} + \alpha_{j} k_{0}^{2} \left| {E_{j}^{2} \left( x \right)} \right|} \right]E_{j} \left( x \right) = 0{\text{ , for j}} = {\text{N}} - {1},{\text{in the}}\,{\text{nonlinear cladding}} $$

5 $$ \left[ {\frac{{d^{2} }}{{dx^{2} }} + q_{j}^{2} + \alpha_{j} k_{0}^{2} \left| {E_{j}^{2} \left( x \right)} \right|} \right]E_{j} \left( x \right) = 0{,}\quad {\text{for j}} = {1},{2},{3} \ldots {\text{N}} - {2},{\text{ in the nonlinear}}\,\,{\text{NRI}}\,\,{\text{slab}}\,\,{\text{guiding}}\,\,{\text{films}}\,\,{\text{and}}\,\,{\text{the}}\,\,{\text{nonlinear interaction}}\,{\text{layers}} $$

6 $$ \left[ {\frac{{d^{2} }}{{dx^{2} }} + q_{j}^{2} + \alpha_{j} k_{0}^{2} \left| {E_{j}^{2} \left( x \right)} \right|} \right]E_{j} \left( x \right) = 0{ , }\,\,\,{\text{for j}} = 0,{\text{ in the nonlinear substrate}} $$

7 $$ q_{j}^{2} = k_{0}^{2} \left( {\varepsilon_{j} \mu_{j} - \beta^{2} } \right){\text{ , for }}\beta < \varepsilon_{{\text{j}}} \mu_{{\text{j}}} \,\,\,{\text{and j}} = 0,{1},{2},{3} \ldots {\text{N}} - {1} $$

8 $$ q_{j}^{2} = Q_{j}^{2} = - k_{0}^{2} \left( {\varepsilon_{j} \mu_{j} - \beta^{2} } \right), {\text{for}}\,\,\beta > \varepsilon_{j} \mu_{j} \,\,{\text{and}}\,\,\,j = 1,3,5 \ldots N - 1 $$

By matching the boundary conditions, the solutions of electric fields in the nonlinear NRI slab guiding films and the nonlinear interaction layers can be written as:

9 $$ E_{j} \left( x \right) = b_{j} cn\left[ {\left. {A_{j} \left( {x + x_{j} } \right)} \right|m_{j} } \right]{ , }\,\,\,\,\,\,{\text{for j}} = {1},{2},{3},...,{\text{N}} - {2},{\text{ in the nonlinearNRIslabguidingfilmsandthenonlinear interactionlayers}} $$

The parameters A_j_, m_j_, x_j_, a_j_^2^, and b_j_^2^ can be expressed as:

10 $$ A_{j} = [q_{j}^{4} + 2\alpha_{j} k_{0}^{2} K_{j} ]^{1/4} ,\,\, m_{j} = \frac{{b_{j}^{2} }}{{a_{j}^{2} + b_{j}^{2} }},\,\,\, x_{j} = cn^{ - 1} \left( {\frac{{E_{j} }}{{b_{j} }}} \right),\,\,a_{j}^{2} = \frac{{\sqrt {q_{j}^{4} + 2\alpha_{j} k_{0}^{2} K_{j} } + q_{j}^{2} }}{{\alpha_{j} k_{0}^{2} }},\,\,\,b_{j}^{2} = \frac{{\sqrt {q_{j}^{4} + 2\alpha_{j} k_{0}^{2} K_{j} } - q_{j}^{2} }}{{\alpha_{j} k_{0}^{2} }} $$

where cn is a specific Jacobian elliptic function, m_j_ is the modulus, and $x_{j}$ is the second constants of integration. By solving the differential Eqs. (4) and (6), the transverse electric fields in the Kerr-type nonlinear cladding and in the Kerr-type nonlinear substrate can be expressed as follows:

11 $$ E_{j} \left( x \right) = E_{j} \left\{ {\cosh [q_{j} \left( {x - x_{j} } \right)] + B_{c} \sinh [q_{j} \left( {x - x_{j} } \right))]} \right\}^{ - 1} { },{\text{ for j}} = {\text{N}} - {1} $$

12 $$ E_{0} \left( x \right) = E_{1} \left\{ {\cosh (q_{0} x) - B_{s} \sinh (q_{0} x)} \right\}^{ - 1} $$

where B_c_ and B_s_ can be expressed as:

13 $$ B_{c} = \sqrt {1 - \frac{{\alpha_{N - 1} E_{N - 1} }}{{2q_{N - 1}^{2} }}} ,\,\,\, B_{s} = \sqrt {1 - \frac{{\alpha_{0} E_{1} }}{{2q_{0}^{2} }}} $$

**Figure 2:**
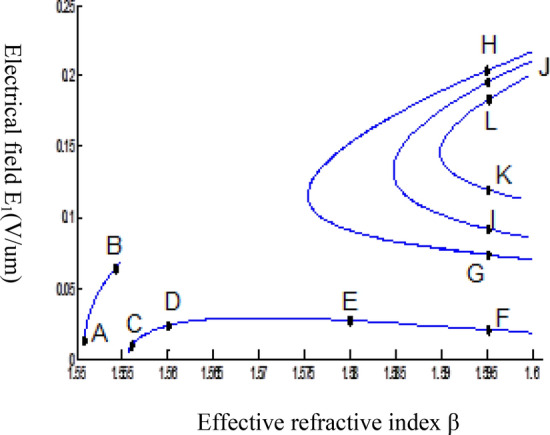
Dispersion curve of the proposed five-layer all Kerr-type nonlinear NRI slab optical waveguide structure with constants: d_f_ = d_1_ = d_3_ = 5 μm and d_i_ = d_2_ = 3 μm.

**Figure 3:**
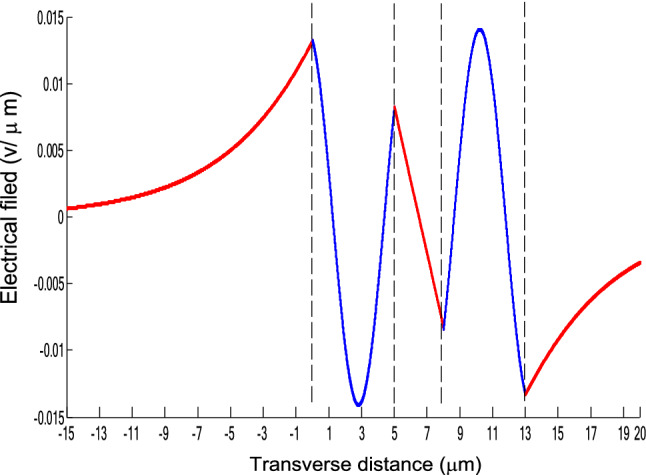
The electrical field distribution of the five-layer all Kerr-type nonlinear NRI slab optical waveguide structure with constants: $\alpha_{0} = \alpha_{1} = \alpha_{2} = \alpha_{3} = \alpha_{4} = 6.3786\,\,\upmu {\text{m}}^{2} /{\text{V}}^{2}$; $\mu_{f} = \mu_{1} = \mu_{3} = - 2$, $d_{1} = d_{3} = 5\,\,\upmu {\text{m}}$, $d_{2} = 3\,\,\upmu {\text{m}}$, $\varepsilon_{f} \mu_{f} = \varepsilon_{1} \mu_{1} = \varepsilon_{3} \mu_{3} = {2}{\text{.4649}}$, $\varepsilon_{0} \mu_{0} = \varepsilon_{2} \mu_{2} = \varepsilon_{4} \mu_{4} = {2}{\text{.4025}}$, and $\lambda = 1.3\,\,\upmu {\text{m}}$ with respect to the point A as shown in Fig. 2.

**Figure 4:**
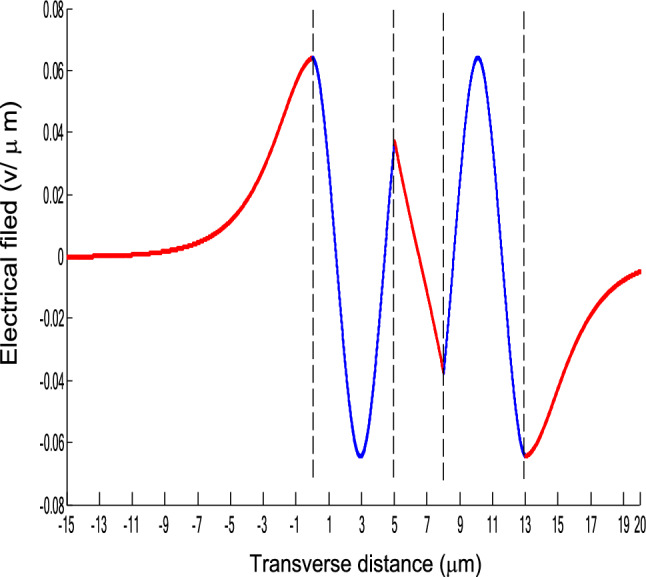
The electrical field distribution of the five-layer all Kerr-type nonlinear NRI slab optical waveguide structure with constants: $\alpha_{0} = \alpha_{1} = \alpha_{2} = \alpha_{3} = \alpha_{4} = 6.3786\,\,\upmu {\text{m}}^{2} /{\text{V}}^{2}$, $\mu_{f} = \mu_{1} = \mu_{3} = - 2$ , $d_{1} = d_{3} = 5\,\,\upmu {\text{m}}$, $d_{2} = 3\,\,\upmu {\text{m}}$, $\varepsilon_{f} \mu_{f} = \varepsilon_{1} \mu_{1} = \varepsilon_{3} \mu_{3} = {2}{\text{.4649}}$, $\varepsilon_{0} \mu_{0} = \varepsilon_{2} \mu_{2} = \varepsilon_{4} \mu_{4} = {2}{\text{.4025}}$, and $\lambda = 1.3\,\,\upmu {\text{m}}$ with respect to the pointB as shown in Fig. 2.

**Figure 5:**
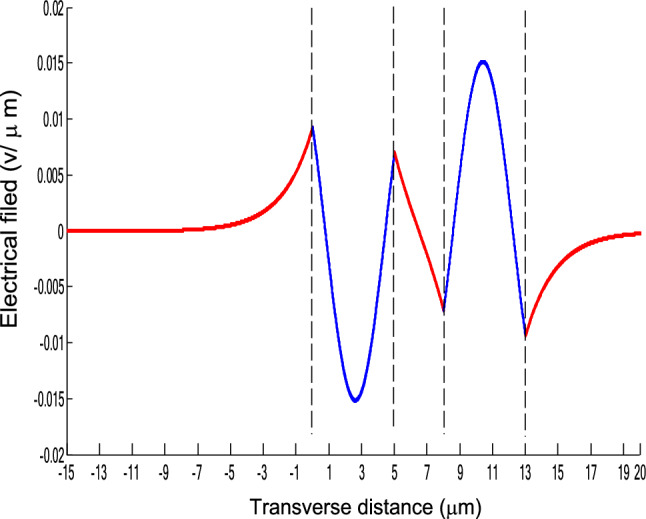
The electrical field distribution of the five-layer all Kerr-type nonlinear NRI slab optical waveguide structure with constants: $\alpha_{0} = \alpha_{1} = \alpha_{2} = \alpha_{3} = \alpha_{4} = 6.3786\,\,\upmu {\text{m}}^{2} /{\text{V}}^{2}$, $\mu_{f} = \mu_{1} = \mu_{3} = - 2$, $d_{1} = d_{3} = 5\,\,\upmu {\text{m}}$, $d_{2} = 3\,\,\upmu {\text{m}}$, $\varepsilon_{f} \mu_{f} = \varepsilon_{1} \mu_{1} = \varepsilon_{3} \mu_{3} = {2}{\text{.4649}}$, $\varepsilon_{0} \mu_{0} = \varepsilon_{2} \mu_{2} = \varepsilon_{4} \mu_{4} = {2}{\text{.4025}}$, and $\lambda = 1.3\,\,\upmu {\text{m}}$ with respect to the point C as shown in Fig. 2.

**Figure 6:**
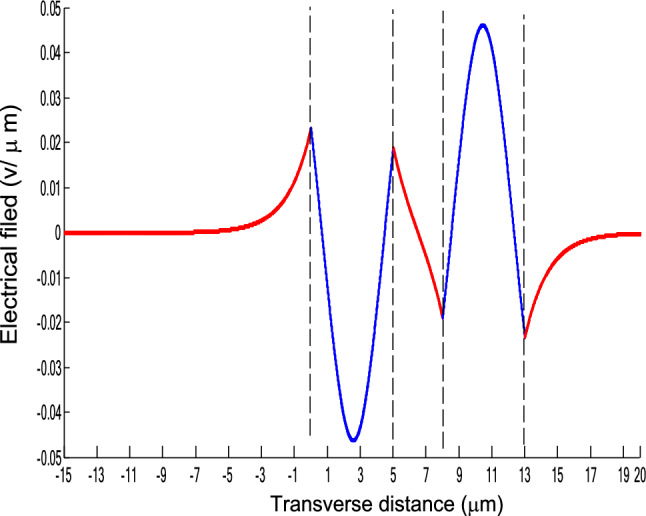
The electrical field distribution of the five-layer all Kerr-type nonlinear NRI slab optical waveguide structure with constants: $\alpha_{0} = \alpha_{1} = \alpha_{2} = \alpha_{3} = \alpha_{4} = 6.3786\,\,\upmu {\text{m}}^{2} /{\text{V}}^{2}$, ${ }\mu_{f} = \mu_{1} = \mu_{3} = - 2$, $d_{1} = d_{3} = 5\,\,\upmu {\text{m}}$, $d_{2} = 3\,\,\upmu {\text{m}}$, $\varepsilon_{f} \mu_{f} = \varepsilon_{1} \mu_{1} = \varepsilon_{3} \mu_{3} = {2}{\text{.4649}}$, $\varepsilon_{0} \mu_{0} = \varepsilon_{2} \mu_{2} = \varepsilon_{4} \mu_{4} = {2}{\text{.4025}}$, and $\lambda = 1.3\,\,\upmu {\text{m}}$ with respect to the point D as shown in Fig. 2.

**Figure 7:**
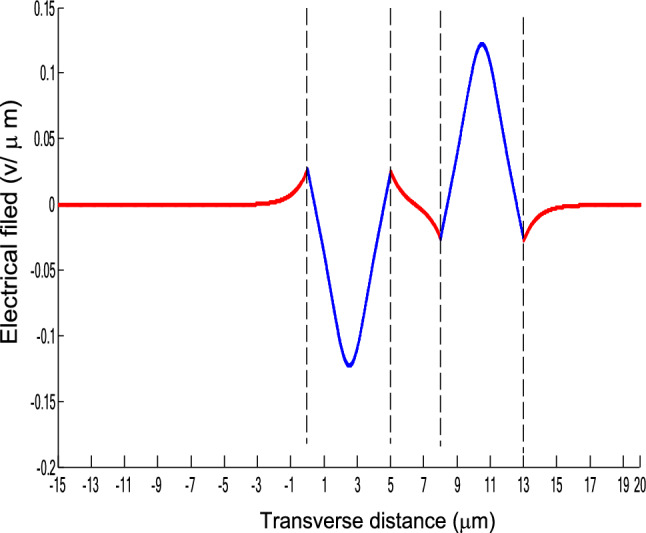
The electrical field distribution of the five-layer all Kerr-type nonlinear NRI slab optical waveguide structure with constants: $\alpha_{0} = \alpha_{1} = \alpha_{2} = \alpha_{3} = \alpha_{4} = 6.3786\,\,\upmu {\text{m}}^{2} /{\text{V}}^{2}$, $\mu_{f} = \mu_{1} = \mu_{3} = - 2$, $d_{1} = d_{3} = 5\,\,\upmu {\text{m}}$, $d_{2} = 3\,\,\upmu {\text{m}}$, $\varepsilon_{f} \mu_{f} = \varepsilon_{1} \mu_{1} = \varepsilon_{3} \mu_{3} = {2}{\text{.4649}}$, $\varepsilon_{0} \mu_{0} = \varepsilon_{2} \mu_{2} = \varepsilon_{4} \mu_{4} = {2}{\text{.4025}}$, and $\lambda = 1.3\,\,\upmu {\text{m}}$ with respect to the point E as shown in Fig. 2.

**Figure 8:**
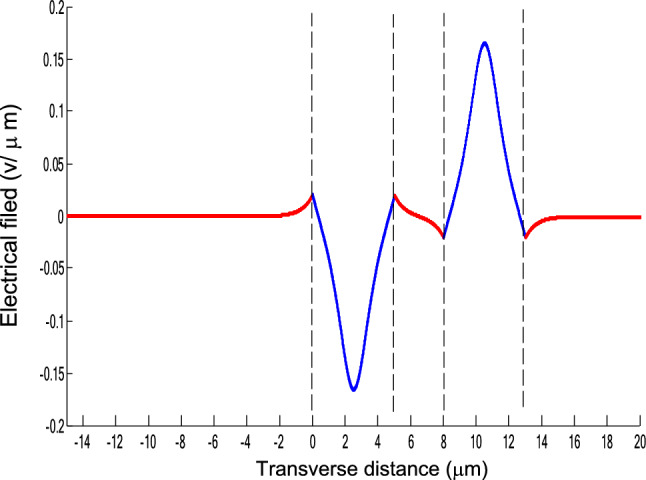
The electrical field distribution of the five-layer all Kerr-type nonlinear NRI slab optical waveguide structure with constants: $\alpha_{0} = \alpha_{1} = \alpha_{2} = \alpha_{3} = \alpha_{4} = 6.3786\,\,\upmu {\text{m}}^{2} /{\text{V}}^{2}$, $\mu_{f} = \mu_{1} = \mu_{3} = - 2$, $d_{1} = d_{3} = 5\,\,\upmu {\text{m}}$, $d_{2} = 3\,\,\upmu {\text{m}}$, $\varepsilon_{f} \mu_{f} = \varepsilon_{1} \mu_{1} = \varepsilon_{3} \mu_{3} = {2}{\text{.4649}}$, $\varepsilon_{0} \mu_{0} = \varepsilon_{2} \mu_{2} = \varepsilon_{4} \mu_{4} = {2}{\text{.4025}}$, and $\lambda = 1.3\,\,\upmu {\text{m}}$ with respect to the point F as shown in Fig. 2.

**Figure 9:**
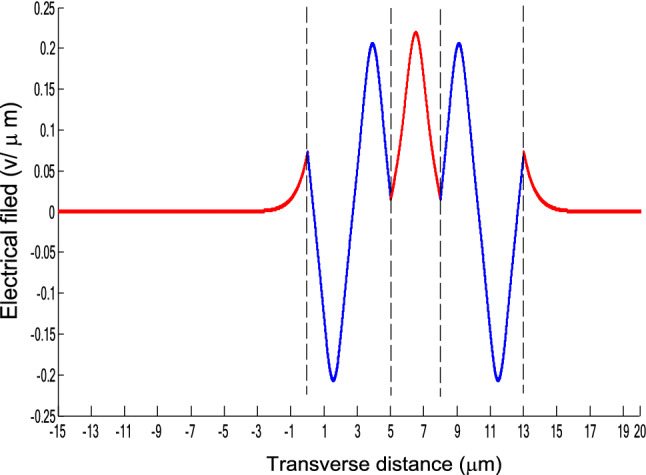
The electrical field distribution of the five-layer all Kerr-type nonlinear metamaterial waveguide with constants: $\alpha_{0} = \alpha_{1} = \alpha_{2} = \alpha_{3} = \alpha_{4} = 6.3786\,\,\upmu {\text{m}}^{2} /{\text{V}}^{2}$, ${ }\mu_{f} = \mu_{1} = \mu_{3} = - 2$, $d_{1} = d_{3} = 5\,\,\upmu {\text{m}}$, $d_{2} = 3\,\,\upmu {\text{m}}$, $\varepsilon_{f} \mu_{f} = \varepsilon_{1} \mu_{1} = \varepsilon_{3} \mu_{3} = {2}{\text{.4649}}$, $\varepsilon_{0} \mu_{0} = \varepsilon_{2} \mu_{2} = \varepsilon_{4} \mu_{4} = {2}{\text{.4025}}$, and $\lambda = 1.3\,\,\upmu {\text{m}}$ with respect to the point G as shown in Fig. 2.

**Figure 10:**
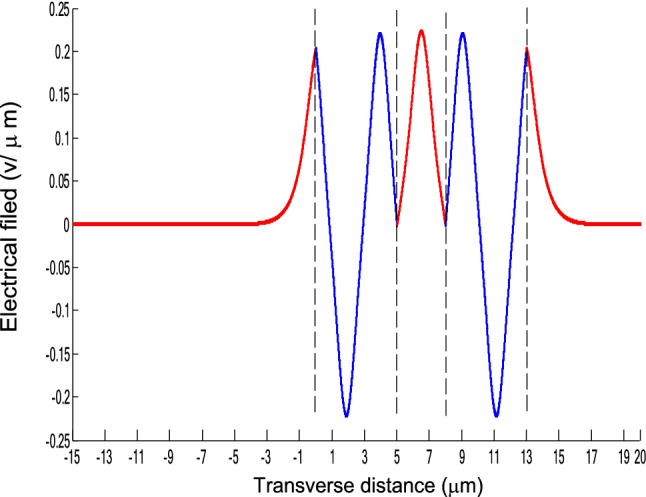
The electrical field distribution of the five-layer all Kerr-type nonlinear NRI slab optical waveguide structure with constants: $\alpha_{0} = \alpha_{1} = \alpha_{2} = \alpha_{3} = \alpha_{4} = 6.3786\,\,\upmu {\text{m}}^{2} /{\text{V}}^{2}$, ${ }\mu_{f} = \mu_{1} = \mu_{3} = - 2$, $d_{1} = d_{3} = 5\,\,\upmu {\text{m}}$, $d_{2} = 3\,\,\upmu {\text{m}}$, $\varepsilon_{f} \mu_{f} = \varepsilon_{1} \mu_{1} = \varepsilon_{3} \mu_{3} = {2}{\text{.4649}}$, $\varepsilon_{0} \mu_{0} = \varepsilon_{2} \mu_{2} = \varepsilon_{4} \mu_{4} = {2}{\text{.4025}}$, and $\lambda = 1.3\,\,\upmu {\text{m}}$ with respect to the point H as shown in Fig. 2.

**Figure 11:**
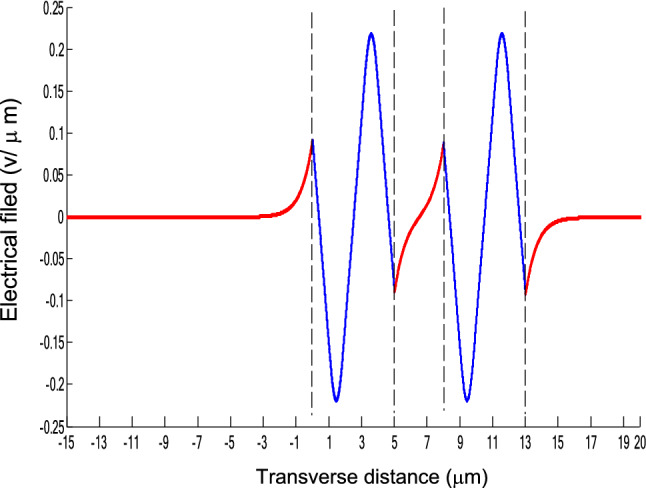
The electrical field distribution of the five-layer all Kerr-type nonlinear NRI slab optical waveguide structure with constants: $\alpha_{0} = \alpha_{1} = \alpha_{2} = \alpha_{3} = \alpha_{4} = 6.3786\,\,\upmu {\text{m}}^{2} /{\text{V}}^{2}$, ${ }\mu_{f} = \mu_{1} = \mu_{3} = - 2$, $d_{1} = d_{3} = 5\,\,\upmu {\text{m}}$, $d_{2} = 3\,\,\upmu {\text{m}}$, $\varepsilon_{f} \mu_{f} = \varepsilon_{1} \mu_{1} = \varepsilon_{3} \mu_{3} = {2}{\text{.4649}}$, $\varepsilon_{0} \mu_{0} = \varepsilon_{2} \mu_{2} = \varepsilon_{4} \mu_{4} = {2}{\text{.4025}}$, and $\lambda = 1.3\,\,\upmu {\text{m}}$ with respect to the point I as shown in Fig. 2.

**Figure 12:**
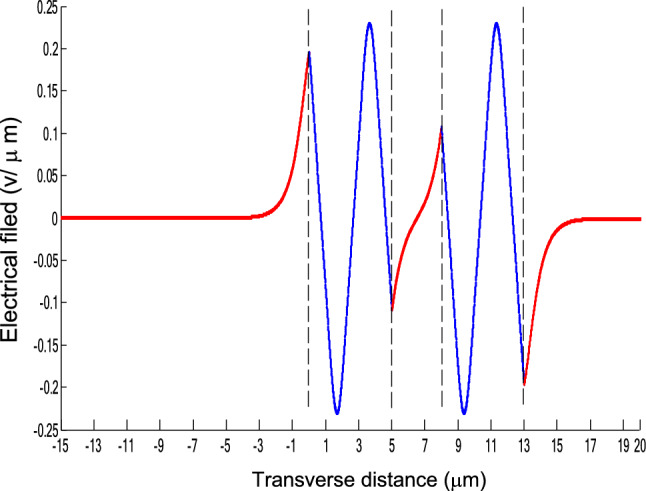
The electrical field distribution of the five-layer all Kerr-type nonlinear NRI slab optical waveguide structure with constants: $\alpha_{0} = \alpha_{1} = \alpha_{2} = \alpha_{3} = \alpha_{4} = 6.3786\,\,\upmu {\text{m}}^{2} /{\text{V}}^{2}$, ${ }\mu_{f} = \mu_{1} = \mu_{3} = - 2$, $d_{1} = d_{3} = 5\,\,\upmu {\text{m}}$, $d_{2} = 3\,\,\upmu {\text{m}}$, $\varepsilon_{f} \mu_{f} = \varepsilon_{1} \mu_{1} = \varepsilon_{3} \mu_{3} = {2}{\text{.4649}}$, $\varepsilon_{0} \mu_{0} = \varepsilon_{2} \mu_{2} = \varepsilon_{4} \mu_{4} = {2}{\text{.4025}}$, and $\lambda = 1.3\,\,\upmu {\text{m}}$ with respect to the point J as shown in Fig. 2.

**Figure 13:**
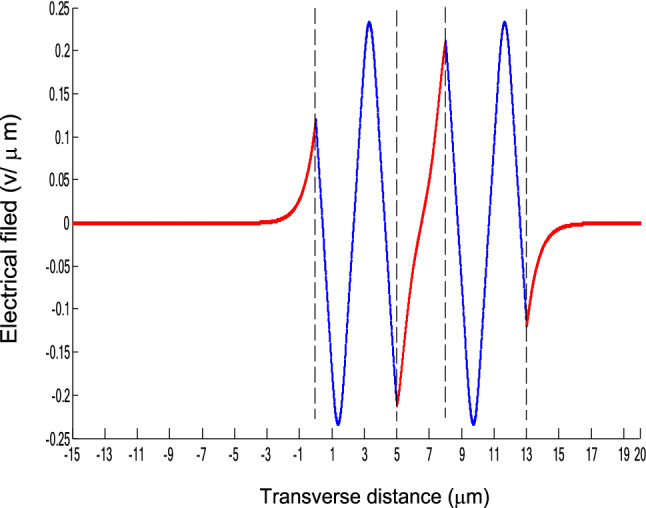
The electrical field distribution of the five-layer all Kerr-type nonlinear NRI slab optical waveguide structure with constants: $\alpha_{0} = \alpha_{1} = \alpha_{2} = \alpha_{3} = \alpha_{4} = 6.3786\,\,\upmu {\text{m}}^{2} /{\text{V}}^{2}$, ${ }\mu_{f} = \mu_{1} = \mu_{3} = - 2$, $d_{1} = d_{3} = 5\,\,\upmu {\text{m}}$, $d_{2} = 3\,\,\upmu {\text{m}}$, $\varepsilon_{f} \mu_{f} = \varepsilon_{1} \mu_{1} = \varepsilon_{3} \mu_{3} = {2}{\text{.4649}}$, $\varepsilon_{0} \mu_{0} = \varepsilon_{2} \mu_{2} = \varepsilon_{4} \mu_{4} = {2}{\text{.4025}}$, and $\lambda = 1.3\,\,\upmu {\text{m}}$ with respect to the point K as shown in Fig. 2.

**Figure 14:**
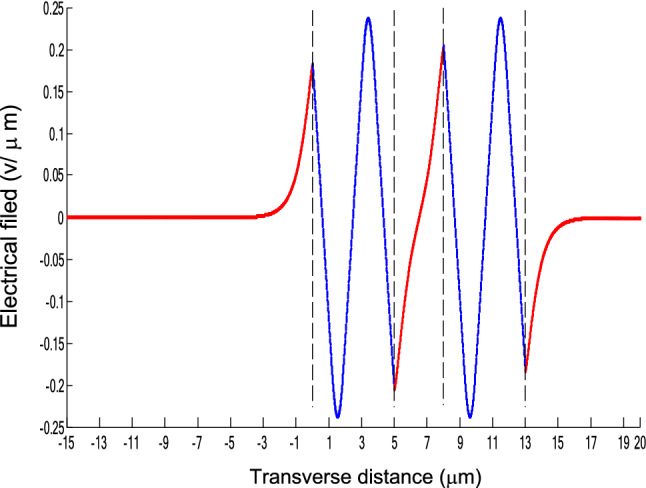
The electrical field distribution of the five-layer all Kerr-type nonlinear NRI slab optical waveguide structure with constants: $\alpha_{0} = \alpha_{1} = \alpha_{2} = \alpha_{3} = \alpha_{4} = 6.3786$ μm^2^/V^2^, ${ }\mu_{f} = \mu_{1} = \mu_{3} = - 2$, $d_{1} = d_{3} = 5\,\,\upmu {\text{m}}$, $d_{2} = 3\,\,\upmu {\text{m}}$, $\varepsilon_{f} \mu_{f} = \varepsilon_{1} \mu_{1} = \varepsilon_{3} \mu_{3} = {2}{\text{.4649}}$, $\varepsilon_{0} \mu_{0} = \varepsilon_{2} \mu_{2} = \varepsilon_{4} \mu_{4} = {2}{\text{.4025}}$, and $\lambda = 1.3\,\,\upmu {\text{m}}$ with respect to the point L as shown in Fig. 2.

The parameters E_1_ and E_N-1_ are the values of the electric fields at the lowest and uppermost boundaries of the nonlinear NRI slab guiding films, respectively. By matching the boundary conditions, the dispersion equation can be written as:

14 $$ E_{j} \left( x \right) = b_{j} cn\left[ {\left. {A_{j} \left( {x + x_{j} } \right)} \right|m_{j} } \right] = E_{{\text{j } - \text{ 1}}} {\text{cn}}\left[ {\left. {A_{j} x_{j} } \right|m_{j} } \right]\frac{{1 - \frac{{{\text{sn}}\left[ {\left. {A_{j} x} \right|m_{j} {\text{]dn}}} \right[\left. {A_{j} x} \right|m_{j} {]}}}{{{\text{cn}}[\left. {A_{j} x} \right|m_{j} {]}}}\varphi_{f} \left[ {A_{j} x_{j} } \right]}}{{1 - m_{j} {\text{sn}}^{2} \left[ {\left. {A_{j} x} \right|m_{j} {\text{]sn}}^{2} } \right[\left. {A_{j} x_{j} } \right|m_{j} {]}}} ,{\text{ for j}} = {1},{ 2},{ 3 } \ldots {\text{ N}} - {2} $$

15 $$ \varphi_{f} \left[ {A_{1} x_{1} } \right] = \frac{{sn\left[ {\left. {A_{1} x_{1} } \right|m_{1} \left] {dn} \right[\left. {A_{1} x_{1} } \right|m_{1} } \right]}}{{cn\left[ {\left. {A_{1} x_{1} } \right|m_{1} } \right]}} = - \frac{{\mu_{1} B_{s} q_{0} }}{{\mu_{0} A_{1} }} $$

16 $$ \frac{{\mu_{j} A_{j + 1} }}{{\mu_{j + 1} A_{j} }} = \frac{{\varphi_{f} [A_{j} d_{j} + A_{j} x_{j} |m_{j} ]}}{{\varphi_{f} [A_{j + 1} x_{j + 1} |m_{j + 1} ]}}{ },\,\,\,\,\,{\text{for j}} = {1},{ 2},{ 3} \ldots {\text{ N}} - {3} $$

17 $$ \begin{aligned}   \frac{{B_{c} q_{{N - 1}} \mu _{j} }}{{\mu _{{N - 1}} A_{j} }} &  = \frac{{\varphi _{f} \left[ {A_{j} d_{j} } \right]\left\{ {dn^{2} \left. {[A_{j} x_{j} } \right|m_{j} \left] { - m_{j} sn^{2} \left. {[A_{{\text{j}}} x_{j} } \right|m_{j} } \right]cn^{2} \left[ {\left. {A_{j} d_{j} } \right|m_{j} } \right]} \right\}}}{{\left[ {1 - \varphi _{f} } \right[A_{j} d_{j} \left] {\varphi _{f} } \right[A_{j} x_{j} ]]\{ 1 - m_{j} sn^{2} \left[ {\left. {A_{j} d_{j} } \right|m_{j} } \right]sn^{2} \left. {[A_{j} x_{j} } \right|m_{j} ]\} }} \\     & \quad  + \frac{{\varphi _{f} \left[ {A_{j} x_{j} } \right]\left\{ {dn^{2} \left[ {\left. {A_{j} d_{j} } \right|m_{j} } \right] - m_{j} sn^{2} \left[ {\left. {A_{j} d_{j} } \right|m_{j} } \right]cn^{2} \left[ {\left. {A_{j} x_{f} } \right|m_{j} } \right]} \right\}}}{{\left[ {1 - \varphi _{f} \left[ {A_{j} d_{j} } \right]\varphi _{f} [A_{j} x_{j} ]} \right]\left\{ {1 - m_{j} sn^{2} \left[ {\left. {A_{j} d_{j} } \right|m_{j} } \right]sn^{2} \left[ {\left. {A_{j} x_{j} } \right|m_{j} } \right]} \right\}}},\quad for\;j = N - 2 \\  \end{aligned}  $$

18 $$ \varphi_{f} \left[ {A_{j} d_{j} } \right] = \frac{{sn\left[ {\left. {A_{j} d_{j} } \right|m_{j} \left] {dn} \right[\left. {A_{j} d_{j} } \right|m_{j} } \right]}}{{cn\left[ {\left. {A_{j} d_{j} } \right|m_{j} } \right]}} $$

19 $$ \varphi_{f} \left[ {A_{j} x_{j} } \right] = \frac{{sn\left[ {\left. {A_{j} x_{j} } \right|m_{j} \left] {dn} \right[\left. {A_{j} x_{j} } \right|m_{j} } \right]}}{{cn\left[ {\left. {A_{j} x_{j} } \right|m_{j} } \right]}} $$

20 $$ \begin{aligned} \varphi_{f} [A_{j} d_{j} + A_{j} x_{j} |m_{j} ] & = \frac{{sn\left[ {A_{j} d_{j} + A_{j} x_{j} \left| {m_{j} \left] {dn} \right[A_{j} d_{j} + A_{j} x_{j} } \right|m_{j} } \right]}}{{cn[A_{j} d_{j} + A_{j} x_{j} |m_{j} ]}} \\ & = \frac{{\varphi_{f} [A_{j} d_{j} |m_{j} ]\{ dn^{2} \left[ {A_{j} x_{j} \left| {m_{j} \left] { - m_{j} sn^{2} } \right[A_{j} x_{j} } \right|m_{j} } \right]cn^{2} [A_{j} d_{j} |m_{j} ]\} }}{{\left\{ {1 - \varphi_{f} \left[ {A_{j} d_{j} \left| {m_{j} \left] {\varphi_{f} } \right[A_{j} x_{j} } \right|m_{j} } \right]} \right\}\left\{ {1 - m_{j} sn^{2} \left[ {A_{j} d_{j} \left| {m_{j} \left] {sn^{2} } \right[A_{j} x_{j} } \right|m_{j} } \right]} \right\}}} \\ & = \frac{{\varphi_{f} [A_{j} x_{j} |m_{j} ]\{ dn^{2} \left[ {A_{j} d_{j} \left| {m_{j} \left] { - m_{j} sn^{2} } \right[A_{j} d_{j} } \right|m_{j} } \right]cn^{2} [A_{j} x_{j} |m_{j} ]\} }}{{\left\{ {1 - \varphi_{f} \left[ {A_{j} d_{j} \left| {m_{j} \left] {\varphi_{f} } \right[A_{j} x_{j} } \right|m_{j} } \right]} \right\}\left\{ {1 - m_{j} sn^{2} \left[ {A_{j} d_{j} \left| {m_{j} \left] {sn^{2} } \right[A_{j} x_{j} } \right|m_{j} } \right]} \right\}}} \\ \end{aligned} $$

where cn, dn, and sn are the Jacobian elliptic functions. Equations (14)–(20) can be solved numerically on a computer. When β and E_1_ are determined, all the other constants A_j_, m_j_, x_j_, q_j_, Q_j_, a_j_, and b_j_ are also determined. The proposed analytic formulas can be used to calculate the transverse electric field function in each layer of the NRI multilayer slab optical waveguide structures. The general formulas can be simplified to analyze five-layer NRI slab optical waveguide structures with all Kerr-type nonlinear layers. Figure 2 shows the dispersion curves of the five-layer all Kerr-type nonlinear NRI slab waveguide structure with the constants:$\alpha_{0} = \alpha_{1} = \alpha_{2} = \alpha_{3} = \alpha_{4} = 6.3786\,$ μm^2^/V^2^, $\mu_{f} = \mu_{1} = \mu_{3} = - 2$, $d_{1} = d_{3} = 5$ μm, $d_{2} = 3$ μm, $\varepsilon_{f} \mu_{f} = \varepsilon_{1} \mu_{1} = \varepsilon_{3} \mu_{3} = {2}{\text{.4649}}$, $\varepsilon_{0} \mu_{0} = \varepsilon_{2} \mu_{2} = \varepsilon_{4} \mu_{4} = {2}{\text{.4025}}$, and $\lambda = 1.3$ μm. Since there always exist a forbidden region near the effective refractive index β = 1.555 for transverse electric waves in the proposed NRI slab waveguide, the points B and C shown in Fig. 2 are not continue. There are five modes in the proposed five layer NRI slab optical waveguide structures with all Kerr-type nonlinear layers at d_f_ = d_1_ = d_3_ = 5 μm and d_i_ = d_2_ = 3 μm. When the points on the same dispersion curve, it means that they belong to the same mode, with the same unique shapes of each mode. For the conventional linear 5-layer slab waveguide structure, the dispersion curve is linear, and no forbidden region exists. When the width of the guiding film increases, the number of the guiding modes will also increase. The direction of the guided power in the proposed five-layer NRI slab optical waveguide structures with all Kerr-type nonlinear layers is opposed to the conventional linear 5-layer slab waveguide structure. Figures 3, 4, 5, 6, 7, 8, 9, 10, 11, 12, 13 and 14 show the electric field distributions of the proposed five-layer NRI slab optical waveguide structures with all Kerr-type nonlinear layers for several points A-L, as shown in Fig. 2. Figure 3 shows the electrical field distribution with respect to the point A, as shown in Fig. 2. Figure 4 shows the electrical field distribution with respect to the point B, as shown in Fig. 2. The points A and B on the same dispersion curve are mode 1. The numerical results show that when the guided power increases, the electric field distributions are gradually narrowed in the Kerr-type nonlinear NRI slab guiding films. The guided power will decrease sharply in the region of Kerr-type nonlinear interaction layer. Figure 5 shows the electrical field distribution with respect to the point C, as shown in Fig. 2. Figure 6 shows the electrical field distribution with respect to the point D, as shown in Fig. 2. Figure 7 shows the electrical field distribution with respect to the point E, as shown in Fig. 2. Figure 8 shows the electrical field distribution with respect to the point F, as shown in Fig. 2. The points C, D, E, and F on the same dispersion curve are mode 2. The numerical results show that when the guided power increases, the electric field distributions are gradually narrowed in the Kerr-type nonlinear NRI slab guiding films. The guided power will decrease in the region of Kerr-type nonlinear interaction layer. Figure 9 shows the electrical field distribution with respect to the point G, as shown in Fig. 2. Figure 10 shows the electrical field distribution with respect to the point H, as shown in Fig. 2. The points G and H on the same dispersion curve are mode 3. The numerical results show that when the guided power increases, the electric field distributions are gradually narrowed in the Kerr-type nonlinear NRI slab guiding films. The guided power will focus in the central region of Kerr-type nonlinear interaction layer. Figure 11 shows the electrical field distribution with respect to the point I, as shown in Fig. 2. Figure 12 shows the electrical field distribution with respect to the point J, as shown in Fig. 2. The points I and J on the same dispersion curve are mode 4. The numerical results show that when the guided power increases, the electric field distributions are gradually narrowed in the Kerr-type nonlinear NRI slab guiding films. The guided power will increase in the region of Kerr-type nonlinear interaction layer. Figure 13 shows the electrical field distribution with respect to the point K, as shown in Fig. 2. Figure 14 shows the electrical field distribution with respect to the point L, as shown in Fig. 2. The points K and L on the same dispersion curve are mode 5. The numerical results show that when the guided power increases, the electric field distributions are gradually narrowed in the Kerr-type nonlinear NRI slab guiding films. The guided power will increase sharply in the region of Kerr-type nonlinear interaction layer.

The proposed general method can also be degenerated to study some special cases:

For case 1, the α_j_ = 0, j = 1, 3, 5…N-2, and $\beta < \varepsilon_{j} \mu_{j}$, A_j_ = q_j_, ${\text{sn}}\left[ {\left. {A_{j} x} \right|m_{j} } \right] = {\sin}\left( {q_{j} x} \right)$,${\text{cn}}\left[ {\left. {A_{j} x} \right|m_{j} } \right] = cos\left( {q_{j} x} \right)$, ${\text{dn}}\left[ {\left. {A_{j} x} \right|m_{j} } \right] = 1$, and the electric field of the Eq. (16) can be rewritten as:

21 $$ E_{j} \left( x \right) = b_{j} cn\left[ {\left. {A_{j} \left( {x + x_{j} } \right)} \right|m_{j} } \right] = A_{f,i} \cos (q_{j} \left( {x - x_{j} } \right)){ },{\text{ for j}} = {1},{ 3},{ 5}...{\text{N}} - {2} $$

For case 2, the α_j_ = 0, j = 1, 3, 5…N-2, and $\beta > \varepsilon_{j} \mu_{j}$, $A_{j}^{2} = Q_{j}^{2} = - k_{0}^{2} \left( {\varepsilon_{j} \mu_{j} - \beta^{2} } \right),{\text{sn}}\left[ {\left. {A_{j} x} \right|m_{j} } \right] = tanh\left( {Q_{j} x} \right)$, ${\text{cn}}\left[ {\left. {A_{j} x} \right|m_{j} } \right] = \sec h\left( {Q_{j} x} \right)$, ${\text{dn}}\left[ {\left. {A_{j} x} \right|m_{j} } \right] = sech\left( {Q_{j} x} \right)$, and the electric field of the Eq. (16) can be rewritten as:

22 $$ E_{j} \left( x \right) = b_{j} cn\left[ {\left. {A_{j} \left( {x + x_{j} } \right)} \right|m_{j} } \right]{ } = A_{f,i} \cosh (Q_{j} \left( {x - x_{j} } \right))\,\,\,{\text{for j}} = {1},{ 3},{ 5 }...{\text{ N}} - {2} $$

For case 3, the parameters α_N-1_ = 0 and B_c_ = 1, the electric field in the nonlinear DPS cladding E_N-1_ can be expressed as:

23 $$ E_{j} \left( x \right) = E_{j} \left\{ {\cosh [q_{j} \left( {x - x_{{\text{N } - \text{ 1}}} } \right)\left] { + B_{c} \sinh [q_{j} \left( {x - x_{N - 1} } \right)} \right]} \right\}^{ - 1} { } = A_{c} \exp ( - q_{j} x){ }\,\,{\text{for j }} = {\text{N}} - {1} $$

For case 4, the parameters α_0_ = 0 and B_s_ = 1, the electric field in nonlinear the substrate E_s_ can be written as:

24 $$ E_{0} \left( x \right) = E_{1} \left\{ {\cosh (q_{0} x) - B_{s} \sinh (q_{0} x)} \right\}^{ - 1} { } = A_{s} \exp (q_{0} x) $$

The Eqs. (21)–(24) can be used to drive some degenerated examples of the NRI multilayer slab optical waveguide structures. The numerical results are same to that of the previous papers^59,60^. It showed that the proposed general method can be degenerated into any kind of the NRI multilayer slab optical waveguide structures. The relative parameters are shown in Online Appendix I–III.

## Conclusions

In this paper, we proposed a general method for analyzing arbitrary NRI multilayer slab optical waveguide structures. This general method can simultaneously be used to degenerate into different kinds of NRI multilayer slab waveguide structures by properly varying the nonlinear coefficient. Some degenerated examples were introduced to prove the accuracy of the proposed method. The analytical and numerical results show excellent agreement. The method can also be degenerated to analyze arbitrary planar conventional optical waveguide structures. Based on this general method, the analysis and calculation of any kinds of NRI multilayer slab optical waveguide structures and conventional multilayer slab optical waveguide structures can be achieved easily.

## Supplementary information

Supplementary file 1
