# Supplementary material for: A general method for analyzing arbitrary planar negative-refractive-index multilayer slab optical waveguide structures
Source: Sci Rep. 2020 Sep 11;10:14964. doi: 10.1038/s41598-020-72017-3 (PMC7486898; doi:10.1038/s41598-020-72017-3)
Supplement: Supplementary file 1 [file 41598_2020_72017_MOESM1_ESM.docx]

^[[1]](#footnote-1)^

**A General Method for Analyzing Arbitrary Planar Negative-refractive-index Multilayer Slab Optical Waveguide Structures**

Yaw-Dong Wu

E-mail: ydwu@nkust.edu.tw

Electronic Engineering Department National Kaohsiung University of Science and Technology

**Appendix I**

Degenerated into a three-layer linear optical NRI slab waveguide:

(i) For $\alpha_{0}=\alpha_{1}=\alpha_{2}=0$, $\beta<\varepsilon_{1}\mu_{1}$, m_1_=0, A_1_=q_1_, $\text{sn}[\left. A_{1}x \right|m_{1}]=\text{sin}(q_{1}x)$, $\text{cn}[\left. A_{1}x \right|m_{1}]=cos(q_{1}x)$, $\text{dn}[\left. A_{1}x \right|m_{1}]=1$, B_c_=1, B_s_=1, $\varepsilon_{f}=\varepsilon_{1}\text{, }\mu_{f}=\mu_{1}\text{, }\mu_{c}=\mu_{0}=\mu_{2}\text{ , }\text{q}_{f}=q_{1}\text{,}\text{q}_{c}=q_{0}=q_{2}$:

$$E_{2}(x)=E_{2}\left\{ \cosh[q_{2}(x-d_{1})]+B_{c}sinh[q_{2}(x-d_{1})] \right\}^{-1}$$

$\text{ }=A_{c}\exp(-q_{2}x),\text{ }x>d_{1}$ (I-1)

$$E_{1}(x)=b_{1}cn[\left. A_{1}(x+x_{1}) \right|m_{1}]$$

$\text{ }=A_{f}cos(q_{1}(x-x_{1}))\text{ , 0}<x<d_{1}$ (I-2)

$$E_{0}(x)=E_{1}\left\{ \cosh(q_{0}x)-B_{s}\sinh(q_{0}x) \right\}^{-1}$$

$\text{ }=A_{s}\exp(q_{0}x),\text{ }x<0$ (I-3)

$\tan(q_{f}d)=\frac{2q_{f}q_{c}\mu_{f}\mu_{c}}{{q_{f}}^{2}{\mu_{c}}^{2}-q_{c}^{2}{\mu_{f}}^{2}}\text{ }$ (I-4)

(ii) For$\alpha_{0}=\alpha_{1}=\alpha_{2}=0$,$\beta>\varepsilon_{1}\mu_{1}$,${A_{1}}^{2}={Q_{1}}^{2}=k_{0}^{2}(\beta^{2}-\varepsilon_{1}\mu_{1})$, $\text{sn}[\left. A_{1}x \right|m_{1}]=tanh(Q_{1}x)$, $\text{cn}[\left. A_{1}x \right|m_{1}]=\sec h(Q_{1}x)$, $\text{dn}[\left. A_{1}x \right|m_{1}]=sech(Q_{1}x)$, $\varepsilon_{f}=\varepsilon_{1}\text{, }\mu_{f}=\mu_{1}\text{, }\mu_{c}=\mu_{0}=\mu_{2}\text{ , }\text{q}_{f}=q_{1}\text{,}\text{q}_{c}=q_{0}=q_{2}$:

$$E_{2}(x)=E_{2}\left\{ \cosh[q_{1}(x-d_{1})]+B_{c}\sinh[q_{1}(x-d_{1})] \right\}^{-1}$$

$\text{ }=A_{c}\exp(-q_{1}x),\text{ }x>d_{1}$ (I-5)

$$E_{1}(x)=b_{1}cn[\left. A_{1}(x+x_{1}) \right|m_{1}]$$

$\text{ }=A_{f}\cosh(Q_{1}(x-x_{1}))\text{ , 0}<x<d_{1}$ (I-6)

$$E_{0}(x)=E_{1}\left\{ \cosh(q_{0}x)-B_{s}\sinh(q_{0}x) \right\}^{-1}$$

$\text{ }=A_{s}\exp(q_{0}x),\text{ }x<0$ (I-7)

$\tanh(Q_{f}d)=\frac{2\mu_{c}\mu_{f}Q_{f}q_{c}}{-{\mu_{c}}^{2}{Q_{f}}^{2}-{\mu_{f}}^{2}{q_{c}}^{2}}$ (I-8)

**Appendix II**

Degenerated into a three-layer optical NRI slab waveguide with Kerr-type nonlinear cladding:

(i) For$\alpha_{0}=\alpha_{1}=0$,$\alpha_{2}\neq0$,$\beta<\varepsilon_{1}\mu_{1}$ , A_1_=q_1_, $\text{sn}[\left. A_{1}x \right|m_{1}]=\text{sin}(q_{1}x)$,$\text{cn}[\left. A_{1}x \right|m_{1}]=cos(q_{1}x)$, $\text{dn}[\left. A_{1}x \right|m_{1}]=1$, B_s_=1, $\varepsilon_{f}=\varepsilon_{1}\text{, }\mu_{f}=\mu_{1}\text{, }\mu_{s}=\mu_{0}, \mu_{c}=\mu_{2}\text{ , }\text{q}_{f}=q_{1}\text{,}\text{q}_{c}=q_{2},\text{ }\text{q}_{s}=q_{0}$:

$E_{2}(x)=E_{2}\left\{ \cosh[q_{2}(x-d_{1})]+B_{c}\sinh[q_{2}(x-d_{1})] \right\}^{-1}\text{, x}>d_{1}$ (II-1)

$$E_{1}(x)=b_{1}cn[\left. A_{1}(x+x_{1}) \right|m_{1}]$$

$\text{ }=A_{f}\cos(q_{1}(x-x_{1}))\text{ , 0}<x<d_{1}$ (II-2)

$$E_{0}(x)=E_{1}\left\{ \cosh(q_{0}x)-B_{s}\sinh(q_{0}x) \right\}^{-1}$$

$\text{ }=A_{s}\exp(q_{0}x),\text{ }x<0$ (II-3)

$\tan(q_{f}d)=\frac{q_{f}B_{c}q_{c}\mu_{f}\mu_{s}+q_{f}q_{s}\mu_{f}\mu_{c}}{{q_{f}}^{2}\mu_{c}\mu_{s}-B_{c}q_{c}q_{s}{\mu_{f}}^{2}}$ (II-4)

(ii) For $\alpha_{0}=\alpha_{1}=0$,$\alpha_{2}\neq0$ , $\beta<\varepsilon_{1}\mu_{1}$, ${A_{1}}^{2}={Q_{1}}^{2}=k_{0}^{2}(\beta^{2}-\varepsilon_{1}\mu_{1})$, $\text{sn}[\left. A_{1}x \right|m_{1}]=tanh(Q_{1}x)$, $\text{cn}[\left. A_{1}x \right|m_{1}]=\sec h(Q_{1}x)$, $\text{dn}[\left. A_{1}x \right|m_{1}]=\sec h(Q_{1}x)$, $\varepsilon_{f}=\varepsilon_{1}\text{, }\mu_{f}=\mu_{1}\text{, }\mu_{s}=\mu_{0}, \mu_{c}=\mu_{2}\text{ , }\text{q}_{f}=q_{1}\text{,}\text{q}_{c}=q_{2},\text{ }\text{q}_{s}=q_{0}$

$E_{2}(x)=E_{2}\left\{ \cosh[q_{2}(x-d_{1})]+B_{c}\sinh[q_{2}(x-d_{1})] \right\}^{-1}\text{, x}>d_{1}$ (II-5)

$$E_{1}(x)=b_{1}cn[\left. A_{1}(x+x_{1}) \right|m_{1}]$$

$\text{ }=A_{f}\cosh(Q_{1}(x-x_{1}))\text{ , 0}<x<d_{1}$ (II-6)

$$E_{0}(x)=E_{1}\left\{ \cosh(x)-B_{s}\sinh(q_{1}x) \right\}^{-1}$$

$\text{ }=A_{s}\exp(q_{1}x),\text{ }x<0$ (II-7)

$\tanh(Q_{f}d)=\frac{\mu_{s}\mu_{f}Q_{f}B_{c}q_{c}+\mu_{c}\mu_{f}Q_{f}q_{s}}{-\mu_{c}\mu_{s}{q_{f}}^{2}-{\mu_{f}}^{2}B_{c}q_{c}q_{s}}$ (II-8)

**Appendix III**

Degenerated into a three-layer optical waveguide with Kerr-type nonlinear NRI slab film:

For $\alpha_{0}=\alpha_{2}=0$, $\alpha_{1}\neq0$, B_c_=1, B_s_=1, $\mu_{f}=\mu_{1}\text{, }\mu_{s}=\mu_{0}, \mu_{c}=\mu_{2}\text{ , }\text{q}_{c}=q_{2},\text{ }\text{q}_{s}=q_{0}, A_{f}=A_{1}, m_{f}=m_{1}$:

$$E_{2}(x)=E_{2}\left\{ \cosh[q_{2}(x-d_{1})]+B_{c}\sinh[q_{1}(x-d_{1})] \right\}^{-1}$$

$\text{ }=A_{c}\exp(-q_{1}x),\text{ }x>d_{1}$ (III-1)

$E_{1}(x)=b_{1}cn[\left. A_{1}(x+x_{1}) \right|m_{1}]\text{ 0}<x<d_{1}$ (III-2)

$$E_{0}(x)=E_{1}\left\{ \cosh(q_{0}x)-B_{s}\sinh(q_{0}x) \right\}^{-1}$$

$\text{ }=A_{s}\exp(q_{0}x),\text{ }x<0$ (III-3)

$\frac{B_{c}q_{c}\mu_{f}}{\mu_{c}A_{f}}=\frac{\varphi_{f}[A_{f}d_{1}]\{dn^{2}\left. [A_{f}x_{f} \right|m_{f}]-m_{f}sn^{2}\left. [A_{f}x_{f} \right|m_{f}]cn^{2}[\left. A_{f}d_{1} \right|m_{f}]\}}{[1-\varphi_{f}[A_{f}d_{1}]\varphi_{f}[A_{f}x_{f}]]\{1-m_{f}sn^{2}[\left. A_{f}d_{1} \right|m_{f}]sn^{2}\left. [A_{f}x_{f} \right|m_{f}]\}}+\frac{\varphi_{f}[A_{f}x_{f}]\{dn^{2}[\left. A_{f}d_{1} \right|m_{f}]-m_{f}sn^{2}[\left. A_{f}d_{1} \right|m_{f}]cn^{2}\left. [A_{f}x_{f} \right|m_{f}]\}}{[1-\varphi_{f}[A_{f}d_{1}]\varphi_{f}[A_{f}x_{f}]]\{1-m_{f}sn^{2}[\left. A_{f}d_{1} \right|m_{f}]sn^{2}\left. [A_{f}x_{f} \right|m_{f}]\}}$ (III-4)

$\varphi_{f}[A_{f}x_{f}]=\frac{sn[\left. A_{f}x_{f} \right|m_{f}]dn[\left. A_{f}x_{f} \right|m_{f}]}{cn[\left. A_{f}x_{f} \right|m_{f}]}=-\frac{\mu_{f}B_{s}q_{s}}{\mu_{s}A_{f}}$ (III-5)

$\varphi_{f}[A_{f}d_{1}]=\frac{sn[\left. A_{f}d_{1} \right|m_{f}]dn[\left. A_{f}d_{1} \right|m_{f}]}{cn[\left. A_{f}d_{1} \right|m_{f}]}$ (III-6)

1. [↑](#footnote-ref-1)
